# Aspartate/asparagine-β-hydroxylase: a high-throughput mass spectrometric assay for discovery of small molecule inhibitors

**DOI:** 10.1038/s41598-020-65123-9

**Published:** 2020-05-26

**Authors:** Lennart Brewitz, Anthony Tumber, Inga Pfeffer, Michael A. McDonough, Christopher J. Schofield

**Affiliations:** Chemistry Research Laboratory, University of Oxford, 12 Mansfield Road, OX1 3TA Oxford, United Kingdom

**Keywords:** Biocatalysis, Chemical libraries, Post-translational modifications, X-ray crystallography, Target validation

## Abstract

The human 2-oxoglutarate dependent oxygenase aspartate/asparagine-β-hydroxylase (AspH) catalyses the hydroxylation of Asp/Asn-residues in epidermal growth factor-like domains (EGFDs). AspH is upregulated on the surface of malign cancer cells; increased AspH levels correlate with tumour invasiveness. Due to a lack of efficient assays to monitor the activity of isolated AspH, there are few reports of studies aimed at identifying small-molecule AspH inhibitors. Recently, it was reported that AspH substrates have a non-canonical EGFD disulfide pattern. Here we report that a stable synthetic thioether mimic of AspH substrates can be employed in solid phase extraction mass spectrometry based high-throughput AspH inhibition assays which are of excellent robustness, as indicated by high Z’-factors and good signal-to-noise/background ratios. The AspH inhibition assay was applied to screen approximately 1500 bioactive small-molecules, including natural products and active pharmaceutical ingredients of approved human therapeutics. Potent AspH inhibitors were identified from both compound classes. Our AspH inhibition assay should enable the development of potent and selective small-molecule AspH inhibitors and contribute towards the development of safer inhibitors for other 2OG oxygenases, e.g. screens of the hypoxia-inducible factor prolyl-hydroxylase inhibitors revealed that vadadustat inhibits AspH with moderate potency.

## Introduction

**Figure 1:**
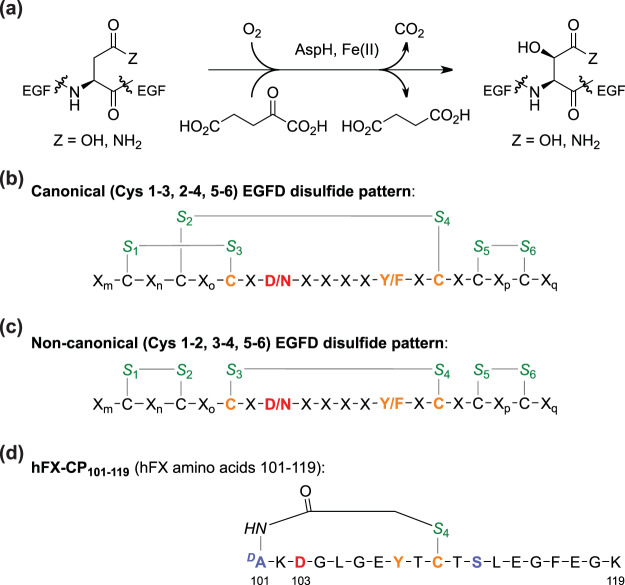
AspH-catalysed substrate hydroxylation. (**a**) General reaction scheme for the AspH-catalysed hydroxylation of Asp/Asn-residues in EGFDs; (**b**) The canonical (Cys 1–3, 2–4, 5–6; green) EGFD disulfide pattern bearing the consensus sequence (orange/red) for AspH-catalysed Asp/Asn-residue (red) hydroxylation; (**c**) The non-canonical (Cys 1–2, 3–4, 5–6; green) EGFD disulfide isomers bearing the consensus sequence (orange/red) for AspH-catalysed Asp/Asn-residue (red) hydroxylation; (**d**) Structure of the cyclic thioether peptide hFX-CP_101–119_. The AspH hydroxylation site (Asp103_hFX_) is in red, consensus sequence residues in orange, cystine sulfurs in green, substituted residues are in light blue; numbering is according to the sequence of human coagulation factor X (hFX).

The human transmembrane protein aspartate/asparagine-β-hydroxylase (AspH, BAH, HAAH) catalyses the hydroxylation of Asp- and Asn-residues in epidermal growth factor-like domains (EGFDs) of its substrates (Fig. 1a)^1,2^. AspH is upregulated in several cancer-types and is reported to be translocated from the endoplasmic reticulum (ER) membrane to the cell surface, a process which is reported to correlate with enhanced tumour invasiveness and poor prognosis^3–6^. However, how the biochemistry of AspH affects tumour cell motility is unknown. Animal models^7–9^ and phenotypes of inherited genetic diseases likely resulting in impaired AspH oxygenase activity (i.e. Traboulsi Syndrome)^10–12^ suggest that AspH activity may regulate the notch signalling pathway. Upregulated AspH is a biomarker^13^ for malign cancers, including hepatocellular carcinoma^14^, glioma^15^, pancreatic cancer^16^, breast cancer^8^, and non-small cell lung carcinoma^17^. AspH is thus an interesting medicinal chemistry target for anticancer therapy.

Strategies to detect and inhibit human AspH have mostly focused on the development of antibodies targeting AspH^5,14,18–21^, antibody drug conjugates^22^, anti-sense mediated RNA knockdown^23,24^, CRISPR-CAS gene knockout^24^, immunotherapy^25^, and vaccination^26^. However, alternative splicing complicates the application of these biological approaches. About ten different isoforms of full-length human AspH are reported with two bearing the catalytically active oxygenase domain^27^; At least one AspH isoform missing the catalytic oxygenase domain is upregulated in cancers^17^.

Comparatively little effort has been directed towards the development of small-molecule AspH inhibitors; this is surprising considering the development of potent and (at least, partially) selective small-molecule inhibitors of human 2OG oxygenases^28,29^. In pioneering cell-based studies, non-selective small-molecule inhibitors of human AspH were identified^30^. In recent years, small-molecules targeting AspH have been employed in cell and animal experiments; however, validation is required to confirm the proposed inhibition mechanism of action of these L-ascorbic acid derivatives^9,31,32^. The development of efficient small-molecule AspH inhibitors for clinical use has been hampered by the lack of efficient assays for isolated recombinant human AspH.

AspH belongs to the class of oxygenases that use 2-oxoglutarate (2OG, α-ketoglutarate) and O_2_ as co-substrates and Fe(II) as a cofactor (Fig. 1a). Like other 2OG oxygenases, AspH has a conserved distorted double-stranded β-helix core fold in its oxygenase domain^33^. The structure of AspH is unique amongst studied human 2OG dependent hydroxylases, as its single Fe(II) cofactor is coordinated by only two protein residues (His679, His725) rather than the typical triad of ligands (HXD/E…H) found in other 2OG dependent protein hydroxylases^33^. The AspH tetratricopeptide repeat (TPR) domain which is adjacent to the AspH oxygenase domain is essential for productive catalysis as it interacts with the EGFD substrates of AspH^34^.

We recently reported the production of soluble truncated recombinant human AspH-constructs, including N-terminally His_6_-tagged AspH_315–758_ (His_6_-AspH_315–758_)^34^. Crystallographic and mass spectrometry (MS)-based experiments unexpectedly indicated that, rather than a canonical EGFD-disulfide connectivity (Cys 1–3, 2–4, 5–6, Fig. 1b), as observed in most crystal and NMR structures of EGFDs^35^, a non-canonical EGFD-disulfide connectivity (Cys 1–2, 3–4, 5–6, Fig. 1c) is necessary for AspH catalysis^34^. In order to avoid disulfide shuffling, stable thioether linked cyclic peptides were designed as synthetic AspH substrates mimicking the central non-canonical Cys 3–4 EGFD fold as essential AspH substrate requirement (Fig. 1d). Using both the His_6_-AspH_315–758_ construct and synthetic AspH substrate cyclic peptides modelled based on the amino acid sequence of reported AspH substrate EGFDs, we developed a mass spectrometry (MS) based assay to monitor its activity^36^. This represented an advance compared to the pioneering assays which used (native) partially purified bovine or murine AspH, monitoring activity by analysing 2OG turnover^37–40^. The utility of the new assay was demonstrated by determining the kinetic properties of AspH; the results indicated that AspH activity may be limited by O_2_ availability^36^, which together with the observation that AspH is upregulated in hypoxia^41,42^, suggests that it might function as an O_2_/hypoxia sensor.

Here we report a robust AspH inhibition assay, which enables evaluation of the effect of small-molecules on AspH activity in a high-throughput manner. Solid phase extraction (SPE) coupled to MS^43–47^ was applied to measure hydroxylation of a cyclic thioether analogue of the non-canonical EGFD disulfide pattern (Fig. 1d). The quality and robustness of the assay was validated by a screening of a compound library of known bioactive small-molecules leading to the identification of novel small-molecule AspH inhibitors. The small-molecule pyridine-2,4-dicarboxylic acid (2,4-PDCA) was identified as a potent AspH inhibitor; a crystal structure of AspH in complex with 2,4-PDCA validates its proposed inhibition mode.

## Results

### Development of a high-throughput AspH inhibition assay

**Figure 2:**
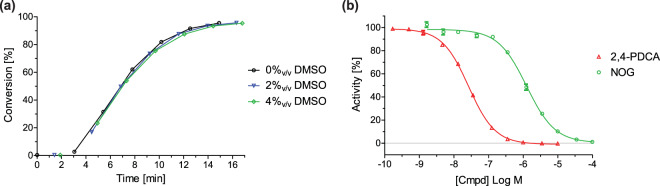
Effect of DMSO and selected small-molecules on AspH activity. (**a**) Monitoring AspH activity in the presence of 0%_v/v_ (black circles), 2%_v/v_ (blue triangles), and 4%_v/v_ DMSO (green diamonds) at time intervals of 1 sample/150 s using SPE-MS; Assay conditions: 50 nM His_6_-AspH_315–758_, 1 μM hFX-CP_101–119_ (Fig. 1d), 100 μM LAA, 3 μM 2OG, and 2 μM FAS, in 50 mM HEPES buffer (pH 7.5, 20 °C); (**b**) Dose-response curves used to determine IC_50_-values for 2,4-PDCA (red triangles) and *N*-oxalylglycine (NOG, green circles). For each compound, one representative set of two independently determined dose-response curves each composed of technical duplicates is shown. IC_50_-values: IC_50_ (2,4-PDCA) = 0.03 ± 0.003 μM; IC_50_ (NOG) = 1.09 ± 0.29 μM; data are reported as a mean of ten (2,4-PDCA) and two (NOG) independent runs (mean ± standard deviation, SD). See Methods Section for details.

We recently reported that AspH activity can be monitored by assaying the hydroxylation of a stable synthetic thioether linked cyclic peptide (hFX-CP_101–119_) based on the EGFD1 sequence of the *in vivo* AspH substrate human coagulation factor X (Fig. 1d)^48,49^. SPE-MS was used to quantify the AspH-catalysed Asp103_hFX_-hydroxylation by monitoring product formation and substrate depletion (+16 Da mass shift)^36^. This SPE-MS based AspH activity assay was modified to evaluate the effect of small-molecules on AspH activity in a high-throughput format. The addition of up to 4%_v/v_ DMSO to the aqueous reaction mixture had no detrimental effect on AspH activity (Fig. 2a). Subsequent determinations of half-maximum inhibitory concentrations (IC_50_) of small-molecules were performed in the presence of 0.5%_v/v_ DMSO using hFX-CP_101–119_-, 2OG-, and Fe(II)-concentrations close to their Michaelis constants (*K*_m_)^36^ in 50 mM HEPES (pH 7.5, 20 °C) under an ambient atmosphere. The reaction was halted whilst in the linear range (7 minutes, ~50% conversion, Fig. 2a) by addition of 10%_v/v_ aqueous formic acid, then analysed by SPE-MS. The assay plate format was changed from 96 to 384 well-plate format to enable parallel determination of IC_50_-values of 16 small-molecules per plate in technical duplicates. The AspH concentration was reduced to 50 nM (enzyme/substrate ratio: 1/20) to minimize consumption of His_6_-AspH_315–758_ and the cyclic hFX-CP_101–119_ substrate peptide (Fig. 1d).

The dose-response curves for the reported non-selective small-molecule AspH inhibitors 2,4-PDCA^30,38^ and *N*-oxalylglycine (NOG)^34^ were then determined using our assay to evaluate their assignment as AspH inhibitors: 2,4-PDCA was confirmed to be a potent small-molecule inhibitor of AspH with its IC_50_-value approaching the lower intrinsic assay limit (IC_50_ = 0.03 ± 0.003 μM; red curve Fig. 2b). NOG^29^ also inhibits AspH activity albeit less efficiently than 2,4-PDCA (IC_50_ = 1.09 ± 0.29 μM; green curve, Fig. 2b).

2,4-PDCA was used as a positive control in all subsequent dose-response experiments; low standard deviations (SD) throughout all measurements with 2,4-PDCA were observed (n = 10). The binding mode of 2,4-PDCA to AspH was then investigated by crystallography, in part to provide evidence that the SPE-MS AspH inhibition assay does not afford false-positive inhibition results.

### Crystallographic validation of the AspH inhibition assay

**Figure 3:**
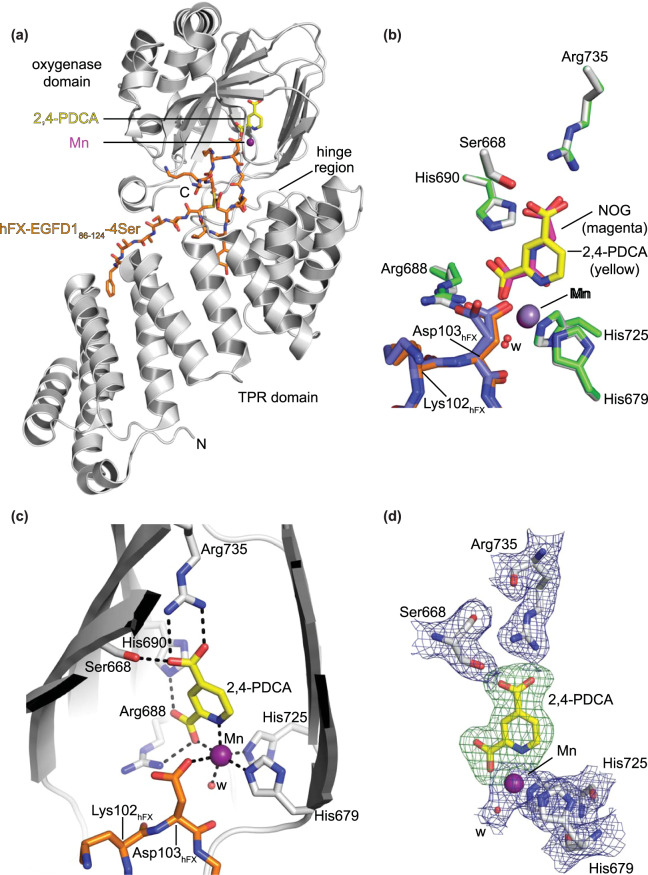
Views from a crystal structure of AspH bound to Mn, 2,4-PDCA, and a synthetic substrate peptide (AspH:2,4-PDCA). Colour code: grey: His_6_-AspH_315–758_; yellow: carbon-backbone of 2,4-PDCA; violet: Mn; orange: carbon-backbone of hFX-EGFD1_86–124_–4Ser peptide; red: oxygen; blue: nitrogen. w: water. (**a**) Overview of the AspH:2,4-PDCA crystal structure; (**b**) Superimposition of active site residues from the AspH:2,4-PDCA (AspH/hFX-EGFD1_86–124_–4Ser/2,4-PDCA: grey/orange/yellow) and AspH:NOG^34^ (AspH/hFX-EGFD1_86–124_–4Ser/NOG: green/slate blue/magenta; PDB entry: 5JQY) crystal structures indicates a similar binding geometry of the AspH active site residues and the small-molecule inhibitors; (**c**) Close-up of the AspH active site: Arg735 forms a salt bridge with the C-4 carboxylate oxygens of 2,4-PDCA (2.4 and 3.1 Å) while Ser668 is positioned to interact with one oxygen atom (2.7 Å). His690 and Arg688 are positioned to interact with the C-2 carboxylate oxygen atoms of 2,4-PDCA (2.8 Å). Mn is bound to His679 (2.3 Å) and His725 (2.2 Å) of His_6_-AspH_315–758_ and coordinates a water molecule (2.2 Å) as well as a C-2 carboxylate oxygen (2.2 Å) and the pyridyl nitrogen atom (2.5 Å) of 2,4-PDCA; (**d**) Electron density (2mF_o_−DF_c_, blue) of Mn, water, and relevant AspH active site residues contoured to 1σ. OMIT electron density map around 2,4-PDCA (mF_o_−DF_c_, green) contoured to 3σ generated by leaving 2,4-PDCA out of the map calculation.

A crystal structure of His_6_-AspH_315–758_ bound to Mn, 2,4-PDCA, and a synthetic AspH substrate peptide (hFX-EGFD1_86–124_–4Ser, Supporting Figure S1) mimicking the full-length EGFD1 of hFX was obtained (AspH:2,4-PDCA, Fig. 3a). His_6_-AspH_315–758_ crystallized in the *P*2_1_2_1_2_1_ space group (2.24 Å resolution), identical to that obtained when using NOG instead of 2,4-PDCA as an inhibitor (AspH:NOG; PDB ID: 5JQY)^34^. The AspH oxygenase domain is comprised of a distorted double-stranded β-helix fold bearing two Fe(II) binding histidine residues;^33,34^ The AspH TPR domain appears to be essential for productive substrate binding as it provides a hydrophobic pocket for conserved EGFD tyrosine or phenylalanine residues in the substrate consensus sequence to bind (Fig. 3a)^34^.

Superimposition of major active site residues from the AspH:2,4-PDCA and AspH:NOG structures shows a high degree of similarity for both AspH (Cα RMSD: 0.19 Å) and the hFX-EGFD1_86–124_–4Ser peptide (Cα RMSD: 0.44 Å): The substrate peptide displays an identical spatial orientation and binding mode. However, the Asp103_hFX_ side chain populates a single conformation in the AspH:2,4-PDCA structure, but adopts two conformations in the AspH:NOG structure (Fig. 3b).

Analysis of the electron density confirms that 2,4-PDCA replaces 2OG and mimics 2OG binding in the AspH active site; it coordinates to the Mn ion, substituting for the Fe(II) cofactor due to crystallisation purposes, in a bidentate fashion by its pyridyl-nitrogen and one of its C-2 carboxylate oxygen atoms (Fig. 3c and d), consistent with other crystal structures of 2OG oxygenases in complex with 2,4-PDCA (Supporting Figures S2 and S3). 2,4-PDCA is positioned to interact via its C-4 carboxylate oxygens with the 2OG C-5 carboxylate binding residues, Arg735 and Ser668 (at least one Lys- or Arg-residue and an alcohol bearing residue are typically involved in 2OG C-5^50^ and 2,4-PDCA C-4 carboxylate binding, Supporting Figure S3). 2,4-PDCA is also positioned to interact with the AspH active site, through its non-metal coordinating C-2 carboxylate oxygen with the *N*^ε^-imidazole nitrogen of His690 (2.8 Å) and its metal coordinating oxygen with a guanidyl nitrogen of Arg688 (2.8 Å); the presence of a non-metal ion chelating histidine at the active site of 2OG oxygenases is not conserved, but is precedented^51^. The C-1 carboxylate of NOG (and likely 2OG) can make an analogous interaction with His690, but the rigid cyclic nature of 2,4-PDCA may favour it on entropic grounds. Overall, analysis of the crystal structures reveals that 2,4-PDCA and NOG bind similarly to AspH by replacing 2OG in the active site rationalizing their activities against AspH and validating the SPE-MS AspH inhibition assay.

### Validation of the robustness of the AspH inhibition assay

**Figure 4:**
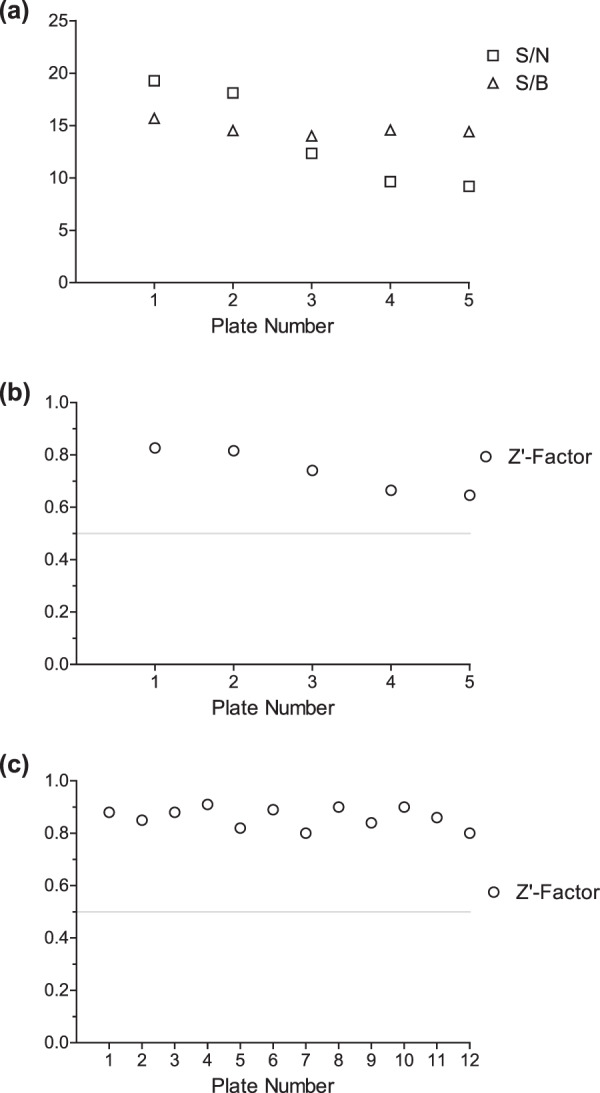
Robustness of the AspH inhibition assay. (**a**) Signal-to-noise ratios (S/N, squares) and signal-to-background ratios (S/B, triangles) of the initial AspH inhibition assay of the five LOPAC assay plates at a fixed inhibitor concentration (20 μM); (**b**) Z’-factors (circles) of the initial AspH inhibition assay of the five LOPAC assay plates at a fixed inhibitor concentration (20 μM); (**c**) Z’-factors for all AspH inhibition assay plates analysed in this publication to determine IC_50_-values (16 compounds per plate including DMSO and 2,4-PDCA as negative and positive controls; technical duplicates in adjacent wells). Z’-factors >0.5 (grey line in b and c) indicate a stable and robust assay of high quality^52^.

Having confirmed that the SPE-MS AspH assay can be applied to identify small-molecule AspH inhibitors, we validated it by profiling AspH against the library of pharmacologically active compounds (LOPAC, Sigma-Aldrich) extended by selected Pfizer-developed bioactive compounds (in total 1370 small-molecules). Initially, the compounds were dispensed on five 384-well plates at a fixed concentration (20 μM), and their inhibition of AspH analysed using the SPE-MS high-throughput assay. The good data quality is reflected by high signal-to-noise (S/N) and signal-to-background (S/B) ratios (Fig. 4a). The five Z’-factors were >0.6 for each plate, indicating, by definition, a highly robust and stable assay of excellent quality (Fig. 4b)^52^.

At a fixed compound concentration (20 μM), 48 out of the 1370 bioactive compounds inhibited >95% AspH activity (see Supporting Table S1 and Supporting Data Sheet). Several of the most potent inhibitors are either flavonoids or contain (metal ion chelating) catechol-moieties and have been identified to inhibit 2OG oxygenases in previous screens^53,54^, including the LOPAC screen^55^. These compounds likely inhibit 2OG oxygenases by reducing free Fe(II) concentration in solution (through chelation), by changing the redox equilibrium *in vitro*, and/or by directly binding the enzymes^53–55^. Out of the initial 48 hits, 14 representative compounds covering the different compound classes were selected to independently determine their dose-responses (IC_50_-values) against AspH (Table 1). All AspH inhibition assays performed to generate dose-response curves were of high quality (Z’-factors >0.5, Fig. 4c).

**Table 1:** Inhibition of AspH by selected small-molecules in the LOPAC screening set.

	AspH-Inhibitor	^a)^Inhibition at 20 µM [%]	^b)^IC_50_ [μM]		AspH-Inhibitor	^a)^Inhibition at 20 µM [%]	^b)^IC_50_ [μM]
**1**		101.7	inactive	**8**		98.1	0.07 ± 0.01
**2**		101.1	2.48 ± 0.14	**9**		97.8	2.45 ± 0.36
**3**		100.8	4.19 ± 0.10	**10**		97.8	0.24 ± 0.01
**4**		100.6	0.51 ± 0.08	**11**		97.4	0.24 ± 0.08
**5**		100.1	^c)^inactive	**12**		96.1	3.53 ± 0.71
**6**		99.9	8.58 ± 1.93	**13**		95.3	7.60 ± 1.95
**7**		99.7	3.41 ± 0.28	**14**		95.2	3.49 ± 0.76

^a)^The complete screening results are shown in the Supporting Data Sheet; ^b)^mean average of two independent runs (n = 2; mean ± SD). AspH inhibition assays were performed as described in the Methods Section using 50 nM His_6_-AspH_315–758_ and 1.0 μM hFX-CP_101–119_ (Fig. 1d) as a substrate; ^c)^candesartan was used in the bioactive acid form rather than as its prodrug cilexetil ester form.

Many flavonoids are part of daily human nutrition and some are taken as health supplements; their impact on modulating human biology is thus of general public health interest. The flavonoid natural product quercetin, which is an ubiquitous part of dietary intake^56,57^ and a reported 2OG oxygenase inhibitor^54^, inhibits AspH activity efficiently (IC_50_ ~ 0.25 μM; Table 1, entry 10). Considering that quercetin uptake has been correlated to inhibit cancer progression^58–60^, it is possible that the inhibition of AspH accounts for some of its biological effect, however, it should be noted that the effects of flavonoids are likely pleiotropic.

The natural product caffeic acid, which is also present in human nutrition and displays a variety of biological effects when administered to humans^61^, inhibits AspH with a similar potency to quercetin (Table 1, entry 11). Caffeic acid is a reported 2OG oxygenase inhibitor;^53,54^ its mechanism of action could either involve displacement of 2OG in the active site, covalent binding to AspH through reaction of its Michael acceptor with nucleophilic protein residues, Fe(II) chelation by its catechol moiety, or modulation of the redox environment by radical scavenging^61^.

Apart from natural products, synthetic bioactive molecules were identified to inhibit AspH catalysis. The potent c-RAF1 kinase inhibitor GW5074^62^, the ionophore calcimycin (A23187)^63^, the inhibitor SCH-202676 effecting agonist and antagonist binding to G-protein-coupled receptors^64^, the leukotriene biosynthesis inhibitor MK-886^65^, and the ion channel antagonist capsazepine^66^ all inhibited AspH activity with SCH-202676 exhibiting the highest potency (IC_50_ ~ 0.5 μM; Table 1, entry 4). The κ-opioid receptor antagonist GNTI^67^ was recognized as potent AspH inhibitor in the initial LOPAC screen (100% inhibition; Table 1, entry 1), but was inactive as a freshly prepared DMSO stock solution, suggesting that a decomposition product may cause the originally observed inhibition.

Several active pharmaceutical ingredients (APIs) approved for human therapy were AspH inhibitors. For instance, the tetracycline-derived antibiotic tigecycline^68^ and sildenafil, the API in Viagra^69^, inhibit AspH with micromolar potency (Table 1, entries 13 and 9). The antibiotic cephalosporin C zinc salt^70^ is a potent AspH inhibitor (IC_50_ ~ 0.07 μM; Table 1, entry 8). The latter observation is likely due to Zn(II) substituting for Fe(II) at the AspH active site, as independently determined dose-response of Zn(II) ions suggest (see below)^38^; other metal-free cephalosporin antibiotics did not inhibit AspH. The anticancer drug PAC-1 activates procaspase-3 by sequestering Zn(II) and presumably inhibits AspH by a similar sequestration of Fe(II)^71^. Candesartan cilexetil, an angiotensin II type 1 receptor antagonist^72^, inhibited AspH activity only in form of its prodrug cilexetil ester, a modification used to increase the bioavailability of the bioactive carboxylic acid drug; no inhibition of the bioactive candesartan containing a free carboxylic acid instead of the cilexetil ester was observed (Table 1, entry 5). This observation likely reflects involvement of the cilexetil ester, which might chelate Fe(II); binding to AspH by candesartan cilexetil was not observed by analyzing the shift of AspH melting temperature (T_m_) in the presence of the small-molecule using differential scanning fluorimetry (ΔT_m_ <0.3 °C, Supporting Figure S4). The structure of benserazide (Ro 4–4602, Table 1, entry 14)^73^, which is administered in combination with levodopa, is similar to catechol-containing compounds and might thus inhibit AspH by chelating Fe(II)^53,54^.

In summary, the LOPAC screen results highlight the quality and robustness of the SPE-MS AspH inhibition assay and led to the identification of both natural product as well as synthetic potent and novel AspH inhibitors. Our assay was then applied to investigate the effects of selected reported 2OG oxygenase inhibitors on AspH activity.

### Inhibition of AspH by reported 2OG oxygenase inhibitors

Clinical interest in modulating the activity of 2OG oxygenases has spurred the development of small-molecule 2OG oxygenase inhibitors, most of which are active site Fe(II) chelators and 2OG competitors; some of which are now APIs in approved human therapeutics^28,29^. A selection of reported 2OG oxygenase inhibitors including various metal salts were profiled against AspH to identify potential lead structures for the development of potent and selective AspH inhibitors and inform on the selectivity, and thus potential side effects, of 2OG oxygenase inhibitors in clinical trials and clinical use.

Initially, the impact of different metal ions on AspH activity was examined, in part because the unusual geometry of the AspH active site suggests that Fe(II) binding to AspH might be different to other 2OG oxygenases as Fe(II) is only bound by two histidine residues (His679, His725; Fig. 3) rather than by the typical three protein ligands (HXD/E…H)^33,34^. Co(II) ions have also been used for treatment of anaemia^74^, in manner that might be mediated via hypoxia-inducible transcription factor (HIF) prolyl hydroxylase inhibition^75,76^. Indeed, Zn(II), Ni(II), and Co(II) inhibited AspH activity, with Zn(II) exhibiting the most pronounced effect (IC_50_ ~ 0.05 μM, Table 2) consistent with observed AspH inhibition by cephalosporin C zinc salt (Table 1, entry 8). Ni(II) and Co(II) are less potent than Zn(II), but still inhibit AspH at sub-micromolar concentrations (Table 2). Inhibition of AspH is reduced approximately 100 fold for Mn(II) compared to Zn(II) (Table 2). Ca(II) ions do not show any effect on AspH activity, at least with the tested thioether substrate hFX-CP_101–119_ (Table 2). The initial results suggest that AspH might be much more sensitive towards the presence of metal ions compared to many other 2OG oxygenases (Table 2), e.g. HIF-α prolyl hydroxylases 1–3 (PHD1–3)^77^, factor inhibiting HIF (FIH)^78^, taurine/αKG dioxygenase (TauD)^79^, and the JmjC lysine-specific demethylases KDM3A^80,81^, KDM4A/JMJD2A^77^, and KDM4E/JMJD2E^77^, though the differences could also relate to the different Fe(II) concentrations and assay techniques used.

**Table 2:** Inhibition (IC_50_) of AspH and selected other 2OG oxygenases by metal ions.

	IC_50_ Zn(II) [μM]	IC_50_ Ni(II) [μM]	IC_50_ Co(II) [μM]	IC_50_ Mn(II) [μM]	IC_50_ Ca(II) [μM]
^a)^AspH	0.05 ± 0.01	0.17 ± 0.08	0.39 ± 0.13	5.06 ± 1.02	inactive
^b)^PHD2^77^	9.3	185	48.3	21	—
^c)^FIH^78^	0.5 ± 0.1	4 ± 1	1.0 ± 0.4	10 ± 1	—
^d)^TauD^79^	—	0.60–1.0	1.0–3.5	—	—
^e)^KDM4A^77^	7.8	9.4	5.7	101.2	—
^e)^KDM4E^77^	13	15.6	9.4	224	—

^a)^Mean average of two independent runs (n = 2; mean ± SD) using 50 nM AspH, 2.0 μM Fe(II), and Zn(OAc)_2_, NiSO_4_, CoCl_2_, MnCl_2_, and CaCl_2_ as metal sources; ^b)^using 2 μM PHD2 and 20 μM F\e(II); ^c)^using 5 μM Fe(II), Ki values are shown; ^d)^using 1 mM TauD and 50 μM Fe(II); ^e)^using 2 μM KDM4 and 10 μM Fe(II).

Subsequently, the broad-spectrum 2OG oxygenase inhibitors IOX1^82^, ebselen^83^, and 2-(4-methylphenyl)-1,2-benzisothiazol-3(2 *H*)-one (PBIT)^54^ were examined in the AspH inhibition assay: these all inhibited AspH activity substantially (IC_50_ < 0.2 μM, Table 3, entries 1–3). Despite their similar IC_50_-values against AspH, IOX1 and PBIT affected the AspH melting temperature differently (ΔT_m_ (IOX1) ~ 2.8 °C; ΔT_m_ (PBIT) ~ −15.2 °C) potentially reflecting different modes of inhibition which might involve covalent binding to AspH for PBIT (Supporting Figure S4). The high potency of IOX1 against AspH is reminiscent of its strong inhibition of the human JmjC lysine-specific demethylases (KDMs) compared to most other tested 2OG hydroxylases^84^.

**Table 3:** Inhibition of AspH by reported 2OG oxygenase inhibitors.

	Inhibitor	^a,b)^IC_50_ [μM]
**1**	IOX1^82^	0.07 ± 0.02
**2**	Ebselen^83^	0.12 ± 0.01
**3**	PBIT^54^	0.14 ± 0.02
**4**	JIB-04^85^	4.24 ± 0.30
**5**	ML324^86^	4.65 ± 1.28
**6**	CPI-455^87^	inactive
**7**	AS-8351^88^	3.07 ± 0.43
**8**	GSK-J1^89,90^	12.89 ± 1.05
**9**	Daminozide^91^	inactive
**10**	TC-E 5002^92^	6.66 ± 0.69
**11**	Deferoxamine mesylate^94^	3.16 ± 0.93
**12**	Deferasirox^96^	2.08 ± 0.81
**13**	Deferiprone^95^	4.89 ± 0.37
**14**	L-Mimosine^97^	5.06 ± 0.28
**15**	Ciclopirox olamine^98,99^	4.39 ± 0.71
**16**	NOFD^100^	15.47 ± 3.13
**17**	Meldonium^101^	inactive
**18**	Roxadustat^102^	19.4 ± 0.7
**19**	Vadadustat^103^	4.39 ± 0.38
**20**	Desidustat^104^	16.30 ± 0.44
**21**	Daprodustat^105^	11.11 ± 1.69
**22**	Molidustat^106^	13.42 ± 1.00

^a)^Mean average of two independent runs (n = 2; mean ± SD). AspH inhibition assays were performed as described in the Methods Section using 50 nM His_6_-AspH_315–758_ and 1.0 μM hFX-CP_101–119_ (Fig. 1d) as a substrate; ^b)^minimum significant ratio (MSR)^109^ = 1.6 (Supporting Figure S5).

Because AspH behaved like a JmjC KDM with respect to IOX1 and to some extent with respect to 2,4-PDCA inhibition^84^, several KDM inhibitors were profiled against AspH (Table 3, entries 4–10): the KDM4 (JMJD2) inhibitors JIB-04^85^ and ML324^86^ inhibit AspH with similar moderate potency. The KDM5A inhibitor CPI-455^87^ was inactive against AspH, while the KDM5B inhibitor AS-8351^88^ inhibits AspH with low micromolar potency (IC_50_ ~ 3.1 μM). GSK-J1, a chemical probe for KDM6^89,90^, is a weak inhibitor of AspH (IC_50_ ~ 12.9 μM); its ester prodrug GSK-J4 was inactive. Both, the plant growth retardant daminozide^91^ and TC-E 5002^92^ are reported KDM2/7 inhibitors; while daminozide is inactive against AspH, TC-E 5002 shows modest potency (IC_50_ ~ 6.7 μM). The inhibition of AspH by some of these compounds should be borne in mind in using them as functional probes in cells and animals.

Three iron chelators are approved by the US food and drug administration (FDA) for treating iron overload in patients suffering from thalassaemia: deferoxamine, deferasirox, and deferiprone^93^. These small-molecules are known inhibitors of 2OG oxygenases^94–96^; they inhibit AspH with IC_50_-values ranging from ~2.0 to 4.9 μM (Table 3, entries 11–13) which is in the range of their IC_50_-values reported for KDM4A, KDM5A, and KDM6B (~3.2 to 17.4 μM)^96^. The natural product L-mimosine^97^ and the FDA-admitted fungicide ciclopirox olamine^98,99^ are structurally related to deferiprone and inhibit AspH, presumably through an analogous mechanism (Table 3, entries 14 and 15).

Other reported (partially) selective 2OG hydroxylase inhibitors including *N*-oxalyl-D-phenylalanine (NOFD), an inhibitor of the Asp/Asn-hydroxylase FIH^100^, or the γ-butyrobetaine dioxygenase (BBOX) inhibitor meldonium (mildronate)^101^, which is in clinical use in some countries, are weak AspH inhibitors (NOFD) or inactive AspH inhibitors (Table 3, entries 16 and 17).

The small molecules roxadustat (FG-4592)^102^, vadadustat (AKB-6548)^103^, desidustat (ZYAN1)^104^, daprodustat (GSK1278863)^105^, and molidustat (BAY85–3934)^106^ inhibit the HIF-α prolyl hydroxylases by chelating the active site Fe(II) or competing with 2OG^107^ and are under clinical investigation, with roxadustat having been approved for treating anemia in patients suffering from chronic kidney disease^76^. These small-molecules are comparably weak AspH inhibitors (Table 3, entries 18–22), with only vadadustat inhibiting AspH with substantial potency (IC_50_ ~ 4.4 μM and ΔT_m_ ~ −0.9 °C, Supporting Figure S4). The latter observation should be considered in clinical application of vadadustat.

## Discussion

The observation that increased levels of AspH on the surface of cancer cells correlates with enhanced tumour invasiveness^3,4,14^ coupled with the observations that AspH is upregulated in many tumours^8,14–17^ and is hypoxically regulated^41,42^, renders AspH an interesting potential cancer target. The exact mechanism of how AspH promotes tumour invasiveness is poorly understood, though there is some evidence it is mediated by the notch receptor and its ligands, which contain multiple EGFDs bearing the consensus sequence for AspH-catalysed hydroxylation^8,9,23,32^. The identification of potent and selective small-molecule AspH inhibitors should help dissect the biological roles of AspH and investigate it as a drug target. However, work towards this has been limited by lack of an efficient AspH assay.

Using a recently reported recombinantly-produced soluble His_6_-AspH_315–758_-construct^34^, we developed an AspH activity assay using SPE-MS to quantify Asp-hydroxylation of a readily accessible and stable cyclic peptide, hFX-CP_101–119_, which mimics the non-canonical Cys 3–4 EGFD disulfide pattern present in AspH substrates (Fig. 1d)^36^. The sensitivity of this method means low levels of both enzyme and substrate are required. Sample preparation is simple as label-free MS-analysis is performed *in situ* resulting in shortened measurement times. Applying the previously determined kinetic parameters of AspH^36^ enabled development of a robust inhibition assay (Fig. 4). NOG and, in particular, 2,4-PDCA were validated as potent AspH inhibitors (Fig. 2b), in accord with prior reports^30,34,38^. In the case of 2,4-PDCA, crystallography defined an active site binding mode analogous to that observed with other 2OG oxygenases (Fig. 3 and Supporting Figures S2 and S3), but identified features (notably interaction with His690) which may be responsible for the unusually potent inhibition of AspH by this 2OG analogue and broad spectrum 2OG oxygenase inhibitor.

Employing the semi-automated high-throughput RapidFire sampling robot, the library of pharmacologically active compounds (LOPAC) was screened, as was done for another 2OG oxygenase, KDM4E (JMJD2E), employing a fluorescence based assay^55^. The stability and robustness of the AspH assay was highlighted by excellent Z’-factors (Fig. 4); the assay only lacked accuracy when strongly ionizing small-molecules suppressed the ionization of the hFX-CP_101–119_ substrate. Both natural products and synthetic bioactive molecules, some of which are APIs of approved human therapeutics, were identified from the LOPAC set as potent AspH inhibitors (Table 1, Supporting Table S1, and Supporting Data Sheet). In general, AspH and KDM4E were inhibited by structurally similar LOPAC compounds, including reported redox-active or metal ion chelators. More compounds were identified that inhibit AspH than KDM4E, possibly reflecting the different assay conditions used (e.g. use of 2 μM Fe(II) for AspH; 10 μM Fe(II) for KDM4E). The potential sensitivity of AspH towards redox active compounds might in part reflect its nature as an ER protein bearing one disulfide and four free cysteine residues in its oxygenase domain^34^. It should be noted that the results of the SPE-MS AspH inhibition assay alone do not define the mechanism of action of the identified AspH inhibitors. Many small-molecules from the obtained LOPAC hit-list likely inhibit AspH by modulating the redox equilibrium of the reaction or by reducing the concentration of available Fe(II). Such compounds can be identified by using a combination of SPE-MS and biophysical techniques such as crystallography (Fig. 3 and Supporting Figures S2 and S3), DSF (Supporting Figure S4), non-denaturing MS, NMR or surface plasmon resonance (SPR)/bio-layer interferometry (BLI) as counterscreens.

The AspH active site geometry is different than that of other human 2OG dependent hydroxylases as the Fe(II) cofactor is bound by only two ligands (His679, His725; Fig. 3) rather than the more typical triad of ligands (HXD/E…H)^33,34^. However, under our assay conditions, the experimentally determined *K*_m_ value of AspH for Fe(II) is in the range of other 2OG oxygenases^36^. Of the tested metal ions, Zn(II) ions inhibited AspH activity with the highest potency (IC_50_ ~ 0.05 μM, Table 2), in agreement with prior studies using native bovine AspH^38^. The inhibitory effect of Zn(II), Ni(II), Co(II), and Mn(II) on AspH activity seems to be enhanced compared to other 2OG oxygenases (Table 2) including the asparaginyl- and aspartyl-residue hydroxylase FIH^78^. Care should be taken in drawing firm conclusions on metal ion binding based on turnover assays, due to differences in conditions employed, e.g. a relatively low Fe(II) concentration was used in our AspH assay and our AspH construct bears a His_6_-tag. Nonetheless, the results suggest relative inhibition of 2OG oxygenases by metal ions may vary in cells to a different degree. This is of biomedical relevance as Co(II) ions have been used to treat anaemia, potentially via inhibition of the HIF prolyl hydroxylases^74–76^.

The presence of Ca(II) ions does not perturb AspH activity under our assay conditions, an observation of potential significance as full-length AspH contains at least one Ca(II)-binding EF hand domain^34^, most AspH substrate EGFDs contain Ca(II)-binding sites around the Asp/Asn-hydroxylation site, and AspH isoforms lacking the catalytic oxygenase domain are involved in cellular Ca(II) homeostasis. However, it should be noted, by contrast with the canonical EGFD domain fold, that the non-canonical disulfide pattern thioether substrate analogue used by us, does not bind Ca(II) ions. Given that the canonical and non-canonical EGFD disulfide forms likely interconvert *in vivo*, the regulation of AspH by Ca(II) ions is a possibility.

Inhibiting human 2OG oxygenases linked to disease by small-molecules is a successful strategy for the development of novel therapeutics^29,33^, as shown by work on inhibition of the HIF prolyl hydroxylases^108^. Some of the JmjC hydroxylases and KDMs are also being investigated as medicinal chemistry targets. Given the roles of many 2OG oxygenases, including AspH, in important biological processes, achieving inhibitor selectivity is likely important, especially when treating long term diseases such as anaemia. We thus tested several APIs of approved and investigated therapeutics against AspH (Table 3). APIs of approved therapeutics such as roxadustat^102^ and meldonium^101^ which presumably inhibit their target 2OG oxygenase by binding to its active site, are weak inhibitors of AspH, likely reflecting structural differences distinguish the binding pockets of their 2OG oxygenase targets (the PHDs for roxadustat and BBOX for meldonium). Among the other APIs currently in clinical trials for treating anemia in patients suffering from chronic kidney disease, only the HIF prolyl hydroxylase inhibitor vadadustat inhibited AspH activity substantially (Table 3, entry 19). The therapeutic APIs deferoxamine, deferasirox, deferiprone, and ciclopirox which exert their biologic function indirectly through the chelation of Fe(II), also inhibit AspH substantially. The results presented here suggest that AspH inhibition should be a consideration in analysing the side effects of the aforementioned APIs.

Overall, the current study should enable the development of selective small-molecule AspH inhibitors which will be useful as chemical probes that will complement biological approaches for inhibiting AspH, as well as helping develop inhibitors selective for other 2OG oxygenases not inhibiting AspH. The natural products and synthetic small-molecules AspH inhibitors identified may be useful as lead structures for initiating AspH inhibitor development programs.

## Methods

### General information

All chemicals were from commercial sources (Sigma-Aldrich, Tocris, Fluorochem, Life technologies) and used as received. Milli-Q ultrapure (MQ-grade) water was used for buffers; LCMS grade solvents were used for solid phase extraction (SPE)-MS. Access to the library of pharmacologically active compounds (LOPAC, Sigma-Aldrich), in form of inhibitor solutions in DMSO, was provided by the Target Discovery Institute, Oxford (the individual compounds are listed in the Supporting Data Sheet). AspH inhibitors identified in the library screen were separately obtained from commercial sources (Sigma-Aldrich, Tocris) to perform dose-response experiments.

### Recombinant AspH production and purification

N-Terminally His_6_-tagged human AspH_315–758_ (His_6_-AspH_315–758_) was produced in *E. coli* BL21 (DE3) cells using a pET-28a(+) vector as previously reported^34,36^. After cell lysis, AspH was purified by Ni(II)-affinity chromatography (HisTrap HP column, GE Healthcare; 1 mL/min flow rate) and size-exclusion chromatography (HiLoad 26/60 Superdex 75 pg 300 mL column; 1 mL/min) using an ÄKTA pure machine (GE Healthcare) as reported. AspH was >95% pure by SDS-PAGE and MS analysis and had the anticipated mass as reported^34^, it was stored in 50 mM HEPES buffer (pH 7.5, 150 mM NaCl) at a concentration of 125 μM at −78 °C; fresh aliquots were used for all biochemical experiments.

### AspH substrates

AspH substrates were designed based on the sequence of EGFD1 of human coagulation factor X (hFX amino acids 86–124)^48,49^; all substrates were prepared with a C-terminal amide. The hFX-EGFD1_86–124_–4Ser peptide was synthesized by solid phase peptide synthesis (SPPS) and purified by GL Biochem (Shanghai) Ltd (Shanghai, China). The thioether linked cyclic peptide hFX-CP_101–119_ (hFX amino acids 101–119) was synthesized from the corresponding linear peptide (D-Ala replacing Cys101_hFX_ and Ser replacing Cys112_hFX_) which was obtained by microwave-assisted SPPS using Fmoc-protection strategy^34,36^.

### AspH inhibition assays

Co-substrate/cofactor stock solutions (L-ascorbic acid, LAA: 50 mM in MQ-grade water; 2-oxoglutarate, 2OG: 10 mM in MQ-grade water; ammonium iron(II) sulphate hexahydrate, FAS, (NH_4_)_2_Fe(SO_4_)_2_·6H_2_O: 400 mM in 20 mM aqueous HCl diluted to 1 mM in MQ-grade water) were freshly prepared every day from commercial solids (Sigma Aldrich).

Solutions of the bioactive small-molecules (100% DMSO) were dry dispensed across 384-well polypropylene assay plates (Greiner) using an ECHO 550 acoustic dispenser (Labcyte). DMSO and 2,4-PDCA were used as negative and positive controls. The DMSO concentration was kept constant at 0.5%_v/v_ throughout all experiments (using the DMSO backfill option of the acoustic dispenser). The initial screening of the LOPAC was performed at a fixed compound concentration (20 μM). For dose-response experiments, the AspH inhibitors were dry dispensed in an approximately three-fold and 11-point dilution series using the acoustic dispenser (100 μM or 10 μM top concentration). 2,4-PDCA was used as a positive control; its IC_50_-value was determined on each assay plate to confirm the assay quality. Each reaction was performed in technical duplicates in adjacent wells on the assay plates; additionally, assays were performed in two independent duplicates on different days using different DMSO inhibitor solutions.

An Enzyme Mixture (25 μL/well), containing 0.1 μM His_6_-AspH_315–758_ in 50 mM HEPES buffer (pH 7.5), was dispensed across the inhibitor-containing 384-well assay plates with a multidrop dispenser (ThermoFischer Scientific) at 20 °C under ambient atmosphere. The plates were subsequently centrifuged (1000 rpm, 30 s) and incubated for 15 minutes. A Substrate Mixture (25 μL/well), containing 2.0 μM hFX-CP_101–119_ (Fig. 1d), 200 μM LAA, 6.0 μM 2OG, and 4.0 μM FAS in 50 mM HEPES buffer (pH 7.5), was added using the multidrop dispenser. Note: The multidrop dispenser ensured proper mixing of the Enzyme and the Substrate Mixtures which was essential for assay reproducibility. The plates were centrifuged (1000 rpm, 30 s) and after incubating for 7 minutes, the enzyme reaction was stopped by addition of 10%_v/v_ aqueous formic acid (5 μL/well). The plates were centrifuged (1000 rpm, 60 s) and analysed by MS.

MS-analyses were performed using a RapidFire RF 365 high-throughput sampling robot (Agilent) attached to an iFunnel Agilent 6550 accurate mass quadrupole time-of-flight (Q-TOF) mass spectrometer operated in the positive ionization mode. Assay samples were aspirated under vacuum for 0.4 s and loaded onto a C4 solid phase extraction (SPE) cartridge. After loading, the C4 SPE cartridge was washed with 0.1%_v/v_ aqueous formic acid to remove non-volatile buffer salts (5 s, 1.5 mL/min). The peptide was eluted from the SPE cartridge with 0.1%_v/v_ aqueous formic acid in 85/15 _v/v_ acetonitrile/water into the mass spectrometer (5 s, 1.25 mL/min) and the SPE cartridge re-equilibrated with 0.1%_v/v_ aqueous formic acid (1 s, 1.25 mL/min). The mass spectrometer parameters were: capillary voltage (4000 V), nozzle voltage (1000 V), fragmentor voltage (365 V), gas temperature (280 °C), gas flow (13 L/min), sheath gas temperature (350 °C), sheath gas flow (12 L/min). The m/z + 2 charge states of the cyclic peptide (substrate) and the hydroxylated cyclic peptide (product) were used to extract ion chromatogram data, peak areas were integrated using RapidFire Integrator software (Agilent). The data were exported into Microsoft Excel and used to calculate the % conversion of the hydroxylation reaction using the equation: % conversion = 100 ×(integral product cyclic peptide) / (integral substrate cyclic peptide + integral product cyclic peptide). Normalized dose-response curves (2,4-PDCA and DMSO controls) were obtained from the raw data by non-linear regression (GraphPad Prism 5) and used to determine IC_50_-values. The standard deviation (SD) of two independent IC_50_ determinations (n = 2) was calculated using GraphPad Prism 5. Signal-to-noise (S/N) and signal-to-background (S/B) ratios^52^ as well as Z’-factors^52^ and minimum significant ratios (MSR)^109^ were calculated according to the cited literature using Microsoft Excel.

### Crystallography

Crystallization experiments were prepared in 96-well, 3-subwell, low profile Intelliplates (Art Robbins Instruments) using a Phoenix RE liquid dispensing robot (Art Robbins Instruments). Crystals were grown using the vapor diffusion method at 4 °C in 200 or 300 nL sitting drops with 2:1, 1:1 or 1:2 sample:well solution ratios and precipitant containing 200 mM NaBr, 20%_v/v_ PEG3350, and 100 mM bis-tris propane at pH 8.5. A His_6_-AspH_315–758_ protein sample (18 mg/mL, 0.33 mM in 50 mM HEPES buffer, pH 7.5) was mixed with neutralized (pH 7.5) 1 mM MnCl_2_, 2 mM 2,4-PDCA, and hFX-EGFD1_86–124_–4Ser peptide (0.726 mmol; AspH/ hFX-EGFD1_86–124_–4Ser: 1/2.2). Crystals were cryo-protected using mother liquor supplemented with 25%_w/v_ glycerol before cryo-cooling in liquid N_2_. Data were collected at 100 K using synchroton radiation at Diamond Light Source (DLS) beamline I04 using a Pilatus 6M-F detector. Data were indexed, integrated, and scaled using the Xia2 strategy of the beamline auto-processing pipeline (Supporting Table S2)^110^.

### Structure solution and refinement

The AspH crystal structure was determined by molecular replacement (MR) using the AutoMR (PHASER^111^) subroutine in PHENIX^112^. The search model used for molecular replacement was based on PDB-ID 5JQY^34^; the structural model was improved by iterative cycles of manual re-building in COOT^113^ and crystallographic refinement in PHENIX (Supporting Table S3).

## Supplementary information

Supplementary information.

Supporting Data.

## Data Availability

The data for the AspH:2,4-PDCA crystal structure are deposited in the protein databank with the PDB accession code 5JTC.
