# Author Correction: Aspartate/asparagine-β-hydroxylase: a high-throughput mass spectrometric assay for discovery of small molecule inhibitors

**DOI:** 10.1038/s41598-020-80008-7

**Published:** 2020-12-21

**Authors:** Lennart Brewitz, Anthony Tumber, Inga Pfeffer, Michael A. McDonough, Christopher J. Schofield

**Affiliations:** Chemistry Research Laboratory, University of Oxford, 12 Mansfield Road, Oxford, OX1 3TA United Kingdom

Correction to: *Scientific Reports*, https://doi.org/10.1038/s41598-020-65123-9, published online 26 May 2020

This Article contains an error in Reference 36, which is incorrectly given as:

Brewitz, L., Tumber, A. & Schofield, C. J. Kinetic parameters of human aspartate/asparagine-β-hydroxylase suggest that it has a possible function in oxygen sensing. *J. Biol. Chem*., https://doi.org/10.1074/jbc.RA1119.012202 (2020).

The correct Reference 36 appears below as Reference 1.
