# Supplementary material for: Aspartate/asparagine-β-hydroxylase: a high-throughput mass spectrometric assay for discovery of small molecule inhibitors
Source: Sci Rep. 2020 May 26;10:8650. doi: 10.1038/s41598-020-65123-9 (PMC7251097; doi:10.1038/s41598-020-65123-9)
Supplement: Supplementary file 1 — Supplementary information. [file 41598_2020_65123_MOESM1_ESM.pdf]

Supporting Information

Aspartate/asparagine-β-hydroxylase: a high-throughput mass spectrometric assay for discovery of small molecule inhibitors

Lennart Brewitz, Anthony Tumber, Inga Pfeffer, Michael A. McDonough, and Christopher J. Schofield*

Chemistry Research Laboratory, University of Oxford, 12 Mansfield Road, OX1 3TA, Oxford, United Kingdom.

Email: christopher.schofield@chem.ox.ac.uk

Supporting Figure S1. The synthetic AspH substrate cyclic peptide hFX-EGFD1$_{86\text{-}124}$-4Ser used in this work. The sequence of the hFX-EGFD1$_{86\text{-}124}$-4Ser peptide is derived from the reported human AspH substrate human coagulation factor X (hFX)[1]. Cystine sulfurs are in green; numbering is according to the sequence of hFX. (1) The canonical (Cys 1–3, 2–4, 5–6) EGFD1 disulfide pattern of hFX bearing the consensus sequence (orange/red) for AspH-catalysed Asp103$_{hFX}$-residue (red) hydroxylation. hFX EGFD1 with the canonical disulfide pattern is not a substrate for isolated AspH; (2) The non-canonical (Cys 1–2, 3–4, 5–6) EGFD1 disulfide pattern of hFX bearing the consensus sequence (orange/red) for AspH-catalysed Asp103$_{hFX}$-residue (red) hydroxylation. The non-canonical hFX EGFD1 is a substrate for isolated AspH[1]; (3) The hFX-EGFD1$_{86\text{-}124}$-4Ser peptide bearing the consensus sequence (orange/red) for AspH-catalysed Asp103$_{hFX}$-residue (red) hydroxylation. The hFX-EGFD1$_{86\text{-}124}$-4Ser peptide is a substrate for isolated AspH[1]. Substituted residues are in light blue.

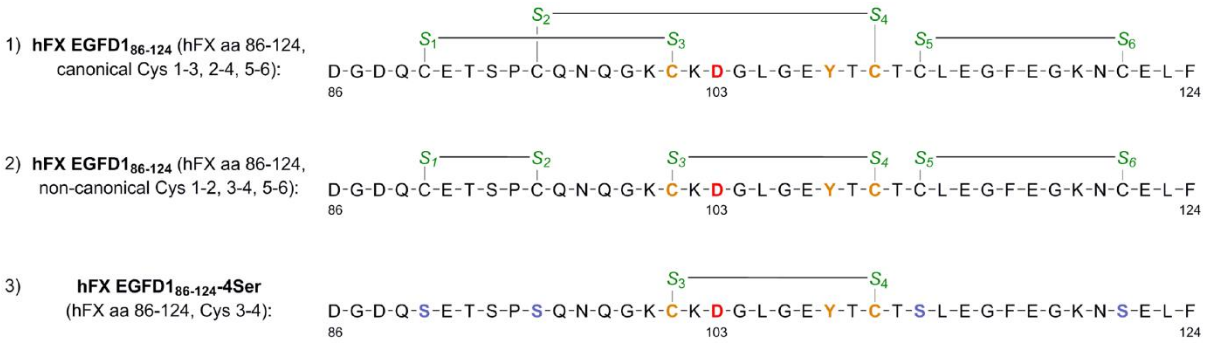

Supporting Figure S2. Comparison of 2OG oxygenase protein substrate complex structures with pyridine-2,4-dicarboxylic acid (2,4-PDCA) bound. Colour code: grey: AspH; yellow: carbon-backbone of 2,4-PDCA; orange: carbon-backbone of the hFX-EGFD1$_{86\text{-}124}$-4Ser peptide (Supporting Figure S1); cyan: lysine-specific demethylase 4B (KDM4B); pink: carbon-backbone of the H3K9me3 peptide; violet: Mn; brown: Ni; red: oxygen; blue: nitrogen. w: water.

In the structures of AspH:2,4-PDCA (**1**; PDB ID: 5JTC) and KDM4B:2,4-PDCA (**2**; PDB ID: 4LXL) the binding of the 2,4-PDCA C-2 and C-4 carboxylates to protein residues differs significantly. In the case of AspH, the 2,4-PDCA C-2 carboxylate is positioned to interact with Arg688$_{\text{AspH}}$ and His690$_{\text{AspH}}$ (both 2.8 Å); the 2,4-PDCA C-4 carboxylate is positioned to form a salt bridge with Arg735$_{\text{AspH}}$ (2.4 and 3.1 Å) and is positioned to interact with Ser668$_{\text{AspH}}$ (2.7 Å). In the case of KDM4B, the 2,4-PDCA C-2 carboxylate is positioned to interact with Lys242$_{\text{KDM4B}}$ (3.0 Å), and faces towards the substrate binding pocket close to the substrate K9me3 group; the 2,4-PDCA C-4 carboxylate is positioned to interact with Tyr133$_{\text{KDM4B}}$ and Lys207$_{\text{KDM4B}}$ (2.6 and 2.7 Å). The spatial orientations of Tyr133$_{\text{KDM4B}}$ and Lys207$_{\text{KDM4B}}$ is apparently determined by interactions with Asn281$_{\text{KDM4B}}$ (3.1 and 2.9 Å). In addition to differences in their binding of 2,4-PDCA, the active sites of AspH and KDM4B differ with regard to both the number of residues interacting with the active site metal and the modes of substrate binding.

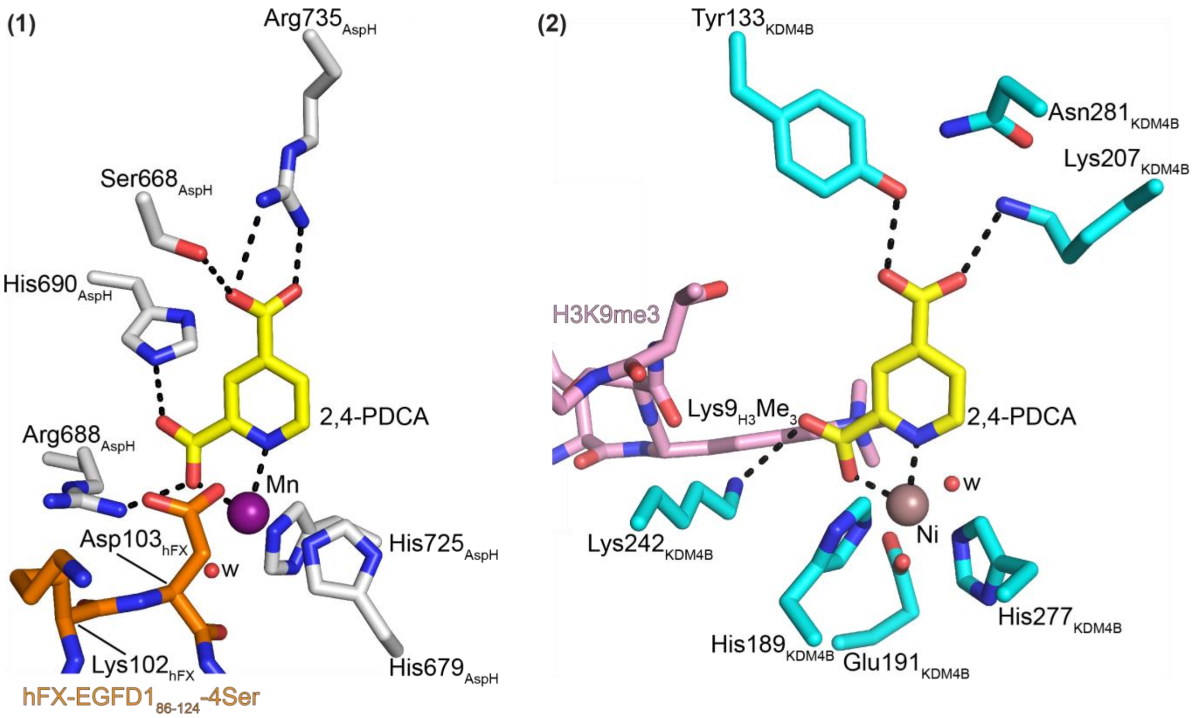

Supporting Figure S3. Comparison of selected human 2OG oxygenase crystal structures in complex with pyridine-2,4-dicarboxylic acid (2,4-PDCA). Colour code: grey: AspH; green: factor inhibiting hypoxia-inducible transcription factor (FIH); salmon: nucleolar protein 66 (NO66); cyan: Jmjc lysine-specific demethylase 4B (KDM4B); slate blue: fat mass- and obesity-associated protein (FTO); magenta: AlkB homolog 5 (Alkbh5); yellow: carbon-backbone of 2,4-PDCA; violet: Mn; orange: Fe; brown: Ni; metallic: Zn; red: oxygen; blue: nitrogen.

Comparison of the AspH:2,4-PDCA (**1**, PDB ID: 5JTC), FIH:2,4-PDCA (**2**, PDB ID: 2W0X)[2], NO66:2,4-PDCA (**3**, PDB ID: 4DIQ), KDM4B:2,4-PDCA (**4**, PDB ID: 4LXL), FTO:2,4-PDCA (**5**, PDB ID: 4IE0)[3], and Alkbh5:2,4-PDCA (**6**, PDB ID: 4NRQ)[4] crystal structures reveals that the precise binding modes of 2OG oxygenases to 2,4-PDCA differ significantly. Between 1 and 3 residues interact directly with the either one or both of the two 2,4-PDCA C-4 carboxylate oxygen atoms; 1 to 2 protein residues interact directly with either one or both of the two 2,4-PDCA C-2 carboxylate oxygen atoms; metal ion coordination sites not occupied by protein residues can be occupied by water molecules (protein ligands and water molecules are not shown for clarity). Knowledge of the different binding modes of 2OG oxygenases with 2,4-PDCA (and related compounds) might be exploited for the design of derivatives that selectively inhibit specific sets of 2OG oxygenases.

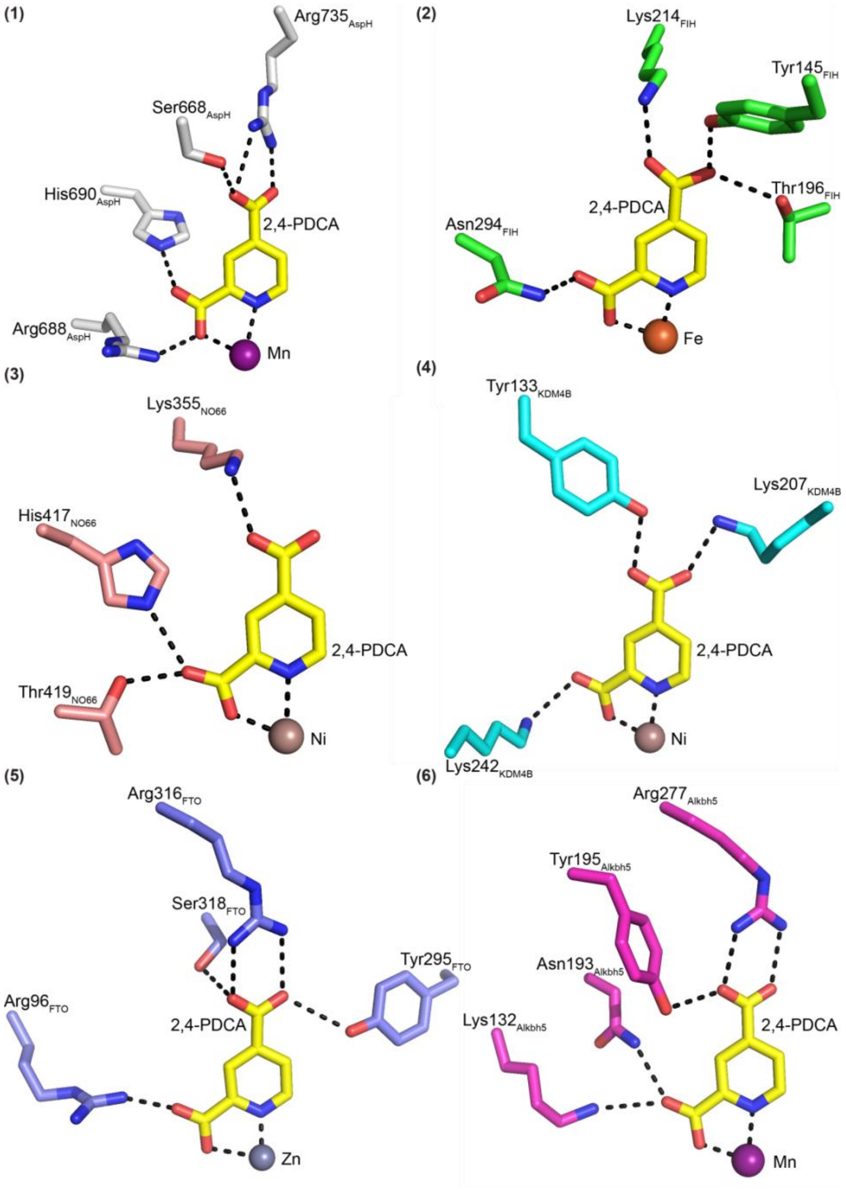

Supporting Table S1. Summary of the 48 small-molecules of the library of pharmacologically active compounds (LOPAC, Sigma-Aldrich) which manifest >95% inhibition of AspH activity at a fixed inhibitor concentration (20 µM).

**AspH-Inhibitor**	**Inhibition** [%]
Aurintricarboxylic acid	103.8
Suramin sodium salt	103.3
6-Hydroxy-DL-DOPA	103.1
Iodoacetamide	102.0
Guanidinyl-naltrindole di-trifluoroacetate	101.7
Morin	101.3
3,5-Dinitrocatechol	101.2
GW5074	101.1
Calcimycin	100.8
Tyrphostin 47	100.6
SCH-202676 hydrobromide	100.6
Tyrphostin 51	100.6
Clodronic acid	100.5
Diethylenetriaminepentaacetic acid	100.3
Candesartan cilexetil	100.1
IPA-3	100.0
Piceatannol	99.9
MK-886	99.9
Capsazepine	99.7
Bisdemethoxycurcumin	99.7
Myricetin	99.4
p-Benzoquinone	99.3
7,8-Dihydroxyflavone hydrate	99.0
Nordihydroguaiaretic acid from Larrea divaricata (creosote bush)	98.9
Tyrphostin AG 835	98.8
(±)-6-Chloro-PB hydrobromide	98.8
Tyrphostin 23	98.7
3,4-Dihydroxyphenylacetic acid	98.6
Caffeic acid phenethyl ester	98.5
Daphnetin	98.5
Hispidin	98.3
Fenoldopam bromide	98.3
Cephalosporin C zinc salt	98.1
Ro 41-0960	97.8
Sildenafil (Viagra)	97.8
Quercetin dihydrate	97.8
1,10-Phenanthroline monohydrate	97.8
(±)-Taxifolin	97.5
Caffeic Acid	97.4
PAC-1	96.1
Apigenin	95.8
(±)-Chloro-APB hydrobromide	95.8
Reactive Blue 2	95.3
Tigecycline	95.3
Benserazide hydrochloride	95.2
SKF 83959 hydrobromide	95.1
R(+)-6-Bromo-APB hydrobromide	95.0
NF 023	94.6

Supporting Figure S4. Differential scanning fluorimetry (DSF) assay. DSF assays were performed in independent duplicates as follows: The assay buffer (50 mM HEPES, pH 7.5, 150 mM NaCl, 50 µM $NiCl_2$) was gently mixed with SYPRO orange (1‰$_{v/v}$) and freshly thawed His_6-$AspH_{315-758}$ was added to a final AspH-concentration of 2 µM. The resulting orange solution was carefully mixed and pipetted into a 96-well Thermoscientific PCR-plate (19 µL per well). Finally, either an inhibitor solution (0.4 mM in DMSO) or a control sample (pure DMSO) was added to each well (1 µL per well, final inhibitor concentration of 20 µM) and the resulting solutions were gently mixed using a pipette. The plate was sealed with an optical tape (Bio-Rag, iCycler iQ), centrifuged (5 sec, 1000 rpm), then placed into a Stratagene Mx3005P PCR machine (Agilent Technologies). The PCR-machine was heated with a rate of 1° C per cycle (starting at an initial temperature of 25 °C; 70 cycles total). Data were analyzed using Microsoft Excel and GraphPad Prism following a literature protocol[5].

Shifts (ΔT_m) of the AspH melting temperature (T_m) are given with respect to DMSO controls. The validated AspH inhibitors 2,4-PDCA and NOG (entries 1 and 2), of which crystal structures in complex with AspH have been reported[1], show a notable increase in the AspH T_m, presumably by stabilizing the AspH active site. The presence of candesartan cilexetil (entry 3) in the assay buffer has no significant effect on the AspH T_m. PBIT (entry 4) seems to strongly destabilize AspH, potentially by binding to one or more of its nucleophilic residues. The positive effect of IOX1 (entry 5) on the AspH T_m is similar to that of NOG, suggesting that crystallization of AspH in the presence of IOX1 could afford co-crystals. Vadadustat seems to slightly destabilize AspH (entry 6).

**Entry**	**AspH-Inhibitor**	[a]**T_m-shift** [°C]
1	2,4-PDCA	3.5 ± 0.6
2	NOG	2.8 ± 0.3
3	Candesartan cilexetil	0.2 ± 0.1
4	PBIT	-15.2 ± 1.4
5	IOX1	2.8 ± 0.2
6	Vadadustat	-0.9 ± 0.2

a) Mean average of two independent runs (n = 2; mean ± standard deviation, SD).

Supporting Figure S5. Minimum significant ratio (MSR) analysis for the AspH inhibitors shown in Table 3. The mean ratio (MR), the MR confidence limits (RLs), the minimum significant ratio (MSR), and the limits of agreement (LsA) were calculated using Microsoft Excel[6,7]. General reproducibility acceptance criteria for assay validation have been reported to be: MSR ≤ 3 and 0.33 < LsA < 3[6]. The MR should ideally be 1 for a reproducible assay, the RLs should include 1[6].

Data of the small-molecule AspH inhibitors displayed in Table 3 (sample size: 22 compounds) have been analysed which is graphically depicted below. The values are in the range of those defined for an acceptable assay: MR = 0.96; RLs = 0.86 and 1.07; MSR = 1.57; LsA = 0.61 and 1.50.

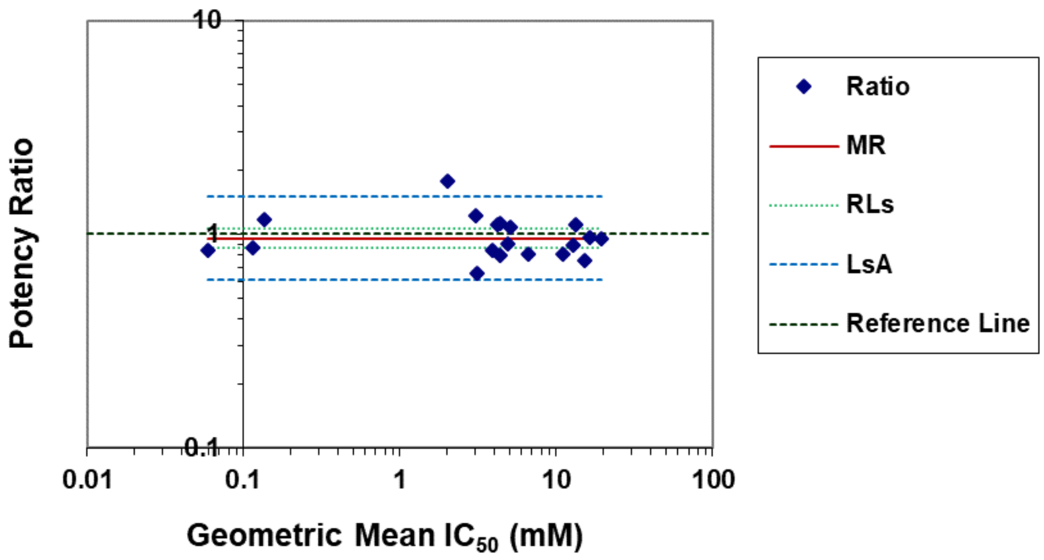

Supporting Table S2. Crystallization conditions and data collection of the AspH:2,4-PDCA complex.

	His_6-$AspH_{315\text{-}758}$·Mn^{II}·2,4-PDCA·hFX-$EGFD1_{86\text{-}124}$-4Ser (AspH:2,4-PDCA)
**PDB ID**	5JTC
**Crystallization**	
Method	Vapor diffusion, sitting drop (200 nL), protein-to-well ratio 1:1
Temperature (K)	277
Crystallization conditions	18 mg/mL His_6-$AspH_{315\text{-}758}$ (330 μM), 1 mM $MnCl_2$, 2 mM pyridine-2,4-dicarboxylic acid, 200 mM NaBr, $20\%_{w/v}$ PEG3350, 100 mM bis-tris propane, pH 8.5, 726 μM hFX-$EGFD1_{86\text{-}124}$-4Ser
AspH:substrate ratio	1:2.2 (330:726 μM)
**Data collection**	
Space group	$P2_12_12_1$
Symmetry	orthorhombic
Cell dimensions:	
a, *b*, *c* (Å)	50.02, 91.66, 123.05
α, β, γ (°)	90.00, 90.00, 90.00
X-Ray source	Synchrotron (Diamond Light Source I04)
Temperature (K)	100
Detector	Pilatus 6M-F
[a)]Resolution (Å)	73.50-2.24 (2.30-2.24)
R_{merge}	0.103 (0.895)
$I / \sigma I$	19.8 (3.2)
Completeness (%)	100.0 (100.0)
Multiplicity	13.1 (13.7)

a) Values in brackets indicate high resolution data shell.

Supporting Table S3. Refinement statistics for the AspH:2,4-PDCA complex.

	His_6-$AspH_{315-758}$·Mn^{II}·2,4-PDCA·hFX-EGFD1_{86-124}-4Ser (AspH:2,4-PDCA)
**PDB ID**	5JTC
**Refinement**	
Resolution (Å)	2.24
No. reflections	27971
R_{work} / R_{free}	0.1963/ 0.2299
No. atoms:	
Protein	3580
Ligand/ion	15
Water	177
B-factors:	
Protein	48.8
Ligand/ion	43.7
Water	45.3
R.m.s. deviations:	
Bond lengths (Å)	0.003
Bond angles (°)	0.749

Supporting References

1. Pfeffer, I. *et al.* Aspartate/asparagine-β-hydroxylase crystal structures reveal an unexpected epidermal growth factor-like domain substrate disulfide pattern. *Nat. Commun.* **10**, 4910 (2019).
2. Conejo-Garcia, A. *et al.* Structural basis for binding of cyclic 2-oxoglutarate analogues to factor-inhibiting hypoxia-inducible factor. *Bioorg. Med. Chem. Lett.* **20**, 6125-6128 (2010).
3. Aik, W. *et al.* Structural basis for inhibition of the fat mass and obesity associated protein (FTO). *J. Med. Chem.* **56**, 3680-3688 (2013).
4. Feng, C. *et al.* Crystal structures of the human RNA demethylase Alkbh5 reveal basis for substrate recognition. *J. Biol. Chem.* **289**, 11571-11583 (2014).
5. Niesen, F. H., Berglund, H. & Vedadi, M. The use of differential scanning fluorimetry to detect ligand interactions that promote protein stability. *Nat. Protoc.* **2**, 2212-2221 (2007).
6. Eastwood, B. J. *et al.* The minimum significant ratio: a statistical parameter to characterize the reproducibility of potency estimates from concentration-response assays and estimation by replicate-experiment studies. *J. Biomol. Screen.* **11**, 253-261 (2006).
7. Haas, J. V., Eastwood, B. J., Iversen, P. W., Devanarayan, V. & Weidner, J. R. Minimum significant ratio – a statistic to assess assay variability. In: Sittampalam GS, Grossman A, Brimacombe K, et al., editors. Assay guidance manual (2017). Bethesda (MD). (Available from: https://www.ncbi.nlm.nih.gov/books/NBK169432/)
